# TyrR is involved in the transcriptional regulation of biofilm formation and D-alanine catabolism in *Azospirillum brasilense* Sp7.

**DOI:** 10.1371/journal.pone.0211904

**Published:** 2019-02-14

**Authors:** Saúl Jijón-Moreno, Beatriz Eugenia Baca, Diana Carolina Castro-Fernández, Alberto Ramírez-Mata

**Affiliations:** Centro de Investigaciones en Ciencias Microbiológicas, Instituto de Ciencias, Benemérita Universidad Autónoma de Puebla., Puebla, Puebla, México; University of Graz, AUSTRIA

## Abstract

*Azospirillum brasilense* is one of the most studied species of diverse agronomic plants worldwide. The benefits conferred to plants inoculated with *Azospirillum* have been primarily attributed to its capacity to fix atmospheric nitrogen and synthesize phytohormones, especially indole-3-acetic acid (IAA). The principal pathway for IAA synthesis involves the intermediate metabolite indole pyruvic acid. Successful colonization of plants by *Azospirillum* species is fundamental to the ability of these bacteria to promote the beneficial effects observed in plants. Biofilm formation is an essential step in this process and involves interactions with the host plant. In this study, the *tyrR* gene was cloned, and the translated product was observed to exhibit homology to TyrR protein, a NtrC/NifA-type activator. Structural studies of TyrR identified three putative domains, including a domain containing binding sites for aromatic amino acids in the N-terminus, a central AAA+ ATPase domain, and a helix-turn-helix DNA binding motif domain in the C-terminus, which binds DNA sequences in promoter-operator regions. In addition, a bioinformatic analysis of promoter sequences in *A*. *brasilense* Sp7 genome revealed that putative promoters encompass one to three TyrR boxes in genes predicted to be regulated by TyrR. To gain insight into the phenotypes regulated by TyrR, a *tyrR*-deficient strain derived from *A*. *brasilense* Sp7, named *A*. *brasilense* 2116 and a complemented 2116 strain harboring a plasmid carrying the *tyrR* gene were constructed. The observed phenotypes indicated that the putative transcriptional regulator TyrR is involved in biofilm production and is responsible for regulating the utilization of D-alanine as carbon source. In addition, TyrR was observed to be absolutely required for transcriptional regulation of the gene *dadA* encoding a D-amino acid dehydrogenase. The data suggested that TyrR may play a major role in the regulation of genes encoding a glucosyl transferase, essential signaling proteins, and amino acids transporters.

## Introduction

In most ecological niches, bacteria are continually exposed to variations in many factors, including nutrient availability. Transcription factors (TFs) induce changes in gene expression that allow bacteria to adapt to such variations, and knowledge of transcriptional regulatory networks is essential for understanding the cellular processes they regulate. The transcription factor TyrR has been shown to play a major role in regulating genes that are essential for the biosynthesis, transport, and degradation of aromatic amino acids [1–3]. In *Escherichia coli*, the tyrosine-responsive regulator TyrR negatively regulates the expression of the tyrosine biosynthesis associated genes *tyrB*, *aroF-tyrA* and *aroLM*, as well as the aromatic amino acid transporter-encoding genes *aroP* and *mtr*, and the *tyrR* gene itself. In addition, TyrR activates the expression of the folate biosynthesis gene *folA* in the presence of tyrosine or phenylalanine [4]. The TyrR regulon has also been studied in two other Enterobacteria, including *Citrobacter freundii*, where it activates the tyrosine degradation gene *tpl* [5], and in *Enterobacter cloacae*, where TyrR regulates the *ipdC* gene, which is involved in the biosynthesis of indole acetic acid (IAA) [6].

*Azospirillum brasilense* is a nitrogen-fixing bacterium that is considered to be a member of the plant-growth promoting rhizobacteria. There is a number of major beneficial effects associated to plants inoculated with *Azospirillum*, including changes in root morphology that result in increases in root elongation, the number of lateral and adventitious roots and the lengthening and branching of root hairs. In addition, the ability to produce phytohormones, primarily IAA and molecules such as nitric oxide has been suggested to underlie the growth response of plants to inoculation by *Azospirillum* species [7]. The principal pathway for IAA biosynthesis involves the intermediate metabolite indol-3-pyruvic acid (IPyA), which is transformed into indole-3-acetaldehyde through a reaction catalyzed by the key enzyme phenylpyruvate decarboxylase, which is encoded by the *ipdC* gene [8, 9].

Bacterial biofilms are surface-associated, multicellular, and morphologically complex microbial communities [10, 11]. The effectiveness of rhizospheric bacteria at promoting plant growth depends on the density these microbes reach in the rhizosphere, in several bacteria, such as *Pseudomonas putida*, *Pseudomonas fluorescens*, *Burkholderia* species, and *Bradyrhizobium japonicum* the quorum sensing regulation is involved in biofilm formation [12, 13], and once a critical microbial density is reached, the biofilm acts as a whole plant growth-promoting unit. The formation of biofilms provides to microorganisms a variety of advantages including, rhizosphere competence and niche-specific adaptation to environmental conditions, allowing for the establishment of an intimate association in which the bacteria attach to the plant root surface. In culture or in association with wheat, the production of exopolysaccharides (EPSs), flocculation, and the transition to cyst-like cells are controlled by *flcA-*encoded response regulator FlcA [14]. *A*. *brasilense* cells are also capable of forming biofilms on both abiotic surface [15, 16] and in association with host plants; when inoculated onto sterile wheat roots, azospirilla colonize the root surfaces extensively, as well as the sites of lateral root emergence [15].

The goal of this study was to assess the possible involvement of the *tyrR*-encoded transcriptional regulator TyrR in IAA biosynthesis. Unexpectedly, TyrR was not observed to be involved in IAA production. However, a genome-wide analysis of *A*. *brasilense* Sp7 resulted in the identification of putative TyrR binding sites in the promoters of genes involved in D-alanine catabolism, regulators of the D-amino acid dehydrogenase (DadA), a putative glucosyl transferase, and putative signaling proteins. Furthermore, we constructed a *tyrR* mutant, and subsequent assays with this strain demonstrated that TyrR is involved in regulating the biofilm formation, the production of EPS, and in regulating the bacterial growth in medium containing D-alanine as the sole carbon source.

## Materials and methods

### Bacterial strains, plasmids, oligonucleotides, and growth conditions

The bacterial strains, plasmids, and oligonucleotides used in this study are shown in Tables 1 and 2. *E*. *coli* strains were grown in LB medium (broth or agar-solidified media) supplemented with 100 μg/mL, ampicillin (Ap); 10 μg/mL, tetracycline (Tc); 30 μg/mL, gentamycin (Gm) and 20 μg/mL kanamycin (Km) as needed. *E*. *coli* was incubated at 37°C for 16 h with shaking (120 rpm), and Bacto agar (Bioxon, Mexico) was added to LB at a concentration of 1.5% to make agar-solidified LB plates. *A*. *brasilense* strains were grown in K-malate minimal medium (broth or agar-solidified media) supplemented with antibiotics (15 μg/mL, Tc or 30 μg/mL, Gm) as needed, and *A*. *brasilense* was incubated at 30°C for 18 h, with shaking (120 rpm). K- malate minimal medium and Congo red (CR) was added to K-malate plates at a final concentration of 40 μg/mL as described previously [15].

**Table 1:** Bacterial strains, and plasmids used in this study.

Strains	Characteristic(s)	Source or reference
*E*. *coli* DH5α	F^-^ *endA1 glnV44 thi-1 recA1 relA1 gyrA96 deoR nupG* Φ80*dlac*ZΔM15 Δ(*lacZYA-argF*)U169, *hsdR*17(*r*_*K*_^-^ *m*_*K*_^+^), λ	Thermo Fisher Scientific, USA
*E*. *coli* S17.1	*recA thi pro hdsR4* (*r*_*K*_^*-*^ *m*_*K*_^*+*^) (RP4-2T::M-Km::Tn7) Tp^r^ Sm^r^ λpir	[17]
*A*. *brasilense* Sp7	Wild-type strain	[18]
*A*. *brasilense* 2112	*A*. *brasilense ipdC*::Sp*-lacZ-* Km^R^ isogenic of Sp7 strain	[8]
*A*. *brasilense* 2116	*A*. *brasilense tyrR*::Gm^R^ isogenic of Sp7 strain	This study
*A*. *brasilense* 2117	*A*. *brasilense tyrR*::Gm^R^ (pJBtyrR)	This study
*A*. *brasilense* 2118	*A*. *brasilense* 2116 (pJB3Tc20)	This study
*A*. *brasilense* 2119	*A*. *brasilense tyrR*::Gm^R^ *ipdC*::Sp*-lacZ-* Km^R^ isogenic of Sp7 strain	This study
**Plasmids**		
pCR2.1-TOPO	Cloning vector, Ap^R^, Km^R^.	Invitrogen Thermo Fisher MX
pBlueScript + (pBSK+)	Cloning vector	Stratagene, La Jolla, CA, USA
pBSL142	Plasmid vector Gm^R^	[19]
pJB3Tc20	Plasmid derived from RK2 oriT, Tc^R^, Ap^R^	[20]
pSUP202	Suicide plasmid Tc^R^, Ap^R^, Cm^R^	[17]
pCR2.1-*tyrR*	Plasmid derived from pCR2.1-TOPO carrying a fragment of 2886 bp with the *tyrR* gene, Ap^R^, Km^R^.	This study
pBSK-*tyrR*-Sp7	Plasmid derived from pBSK+ carrying the *tyrR* gene in a fragment of 1964 bp	This study
pCR2.1-*tyrR*::Gm^R^	Plasmid derived from pCR2.1-*tyrR*, with *tyrR*::Gm^R^	This study
pAB2116	Plasmid derived from pSUP202 carrying *tyrR*::Gm^R^ in a fragment of 3.5 kb	This study
pJB3-*tyrR*-Sp7	Plasmid derived from pJB3Tc20 carrying the *tyrR* gene in a fragment of 1542 bp	This study

Gm = Gentamycin, Ap = Ampicillin, Tc = Tetracycline, Cm = Chloramphenicol, Km = Kanamycin.

**Table 2:** Oligonucleotides used in this study.

Oligonucleotides	Characteristic(s)	Source or reference
TyrR-F1	5 ʹAACGGCTTCACCAGCGTCGG 3 ʹ	This study
TyrR-R1.1	5 ʹ GGATGGGTGCGCCTGGTTCC 3 ʹ	This study
RGm3	5 ʹ GGGAAATCGATCTCGGCTTGAACG 3 ʹ	This study
F-tyrR-Sp7	5 ʹ CCACCACCGTGACCTCGTG 3 ʹ	This study
TyrR-R1	5´CGCGGAGGGCCATTTCAGCA 3´	This study
PrTyrR-F-Sp7	5 ʹ CACCATGCGCATCGACGTCCT 3 ʹ	This study
PrTyrR-R-Sp7	5 ʹ GGTCCCGTCCGGAATCCCATAC 3 ʹ	This study
tyrR-RT-F	5 ʹ CGAGATCGGGGAGATGTCG 3 ʹ	This study
tyrR-RT-R	5 ʹ CAGCACGTTCAGTGG 3 ʹ	This study
dadA-RT-F	5 ʹ CAGGTCTTCCGTACCCAGAA 3 ʹ	This study
dadA-RT-R	5 ʹ CGATCTTCTCCTTGACCAGC 3 ʹ	This study
gyrA-RT-F	5 ʹ TCACCGACGAAGAGTTGATG 3 ʹ	[21]
gyrA-RT-R	5 ʹ CTCTTCGATCTCGGTCTTGG 3 ʹ	[21]

### Amplification, cloning and sequencing of the *tyrR* gene from *A*. *brasilense* Sp7

The purification of genomic and plasmid DNA used for DNA restriction enzyme digestion, electrophoretic agarose analysis, and transformation assays were carried out according to standard protocols [22]. The *tyrR* gene was obtained by PCR amplification using the primers TyrR-F1 and TyrR-R1.1 (Table 2), Platinum Taq DNA polymerase High Fidelity (Invitrogen, Thermo Fisher Scientific), and genomic DNA from the Sp7 strain. The 2.9 kb amplicon included the *tyrR* gene (GenBank accession number AMK58_07015) and 285 bp downstream of the *tyrR* gene. Next, the amplicon was cloned in pCR2.1 TOPO (Invitrogen, Thermo Fisher Scientific) to yield pCR2.1-*tyrR* and was transformed into *E*. *coli* DH5α competent cells. The fragment was sequenced at the Sequencing Unit at the Universidad Nacional Autonoma de Mexico (UNAM).

### Structural analysis of the TyrR protein

The I-Tasser server was used for automated protein structure and function predictions [23, 24]. Protein domain sequence comparisons were performed for the well-characterized N- terminus of the TyrR protein of *E*. *coli* (PDB accession number 2JHE) [25], the central AAA**⁺** domain of the *Pseudomonas aeruginosa* protein FleQ (PDB accession number 5EXP) [26], and the C- terminal helix-turn-helix DNA binding motif from the *Haemophilus influenzae* TyrR protein (PDB accession number 1G2H) [27] were used for the structural analyses as suggested by the server, and the I-Tasser parameters were used. In this study, models exhibiting higher C-scores (better model) were used, and models analyzed against the structure exhibiting a higher resolution were used to create the corresponding model. The analysis was performed, and the figures were made using the UCSF Chimera program [28]. In addition, alignments were performed using Clustal Omega with the following sequences obtained from BLASTP: FleQ from *P*. *aeruginosa* (accession number AAC37124), TyrR from *A*. *brasilense* Sp7 (accession number WP_035673475), TyrR from *E*. *coli* K12 (accession number EG11042), and TyrR from *E*. *cloacae* (accession number ACB55419). Logos were generated using Weblogo 3 Create [29].

### Construction of *A*. *brasilense* 2116, 2117, 2118, and 2119 strains

The chromosomal region in *A*. *brasilense* Sp7 corresponding to the *tyrR* gene was mutagenized by insertion of a Gm resistance cassette gene via homologous recombination (S1 Fig). A plasmid carrying the *tyrR*::Gm^R^ construct was generated by digesting the plasmids pCR2.1-*tyrR* and pBSLl42 with *Sal*I. The *Sal*I fragment containing the Gm cassette from pBSLl42 was ligated into the *Sal*I site of pCR2.1-*tyrR* to obtain the plasmid pCR2.1-*tyrR*::Gm^R^, which was transformed in *E*. *coli* DH5α competent cells. Next, a 3.5 kb amplicon was obtained by PCR using the primers TyrR-F1 and TyrR-R1.1 and Platinum Taq DNA polymerase High Fidelity (Invitrogen), which was subsequently cloned into the *Eco*RV site of the suicide plasmid pSUP202 [17] to generate the plasmid pAB2116, which was transformed into *E*. *coli* S17.1 [17] competent cells. Conjugation between *E*. *coli* S17 (pAB2116) and *A*. *brasilense* was carried out according to the method described by Carreño-López *et al*. [8]. To assess whether the correct replacement event had occurred, the resulting *A*. *brasilense* transconjugants were screened by PCR using the primer sets TyrR-F1 and TyrR-R1.1, TyrR-F1 and RGm3, and PrTyrR-F-Sp7 and PrTyrR-R-Sp7.

The *tyrR* gene was PCR amplified from *A*. *brasilense* Sp7 chromosomal DNA using the primers F-tyrR-Sp7 and TyrR-R1. The resulting 1,964 bp fragment, which contained the native promoter and the *tyrR* ORF was cloned into pBSK+ previously digested with the *Eco*RV restriction enzyme. The pBSK-*tyrR-*Sp7 and pJB3Tc20 [20] plasmids were both digested with the restriction enzymes *Hind*III and *Bam*HI, and the 1,964 bp fragment was subsequently ligated to the yield pJB3-*tyrR*-Sp7 plasmid, which was then transformed in *E*. *coli* S17.1 competent cells. Next, the *E*. *coli* S17.1(pJB3-*tyrR-*Sp7) strain was used as a donor to transfer the pJB3*-tyrR*-Sp7 plasmid to 2116 recipient strain as described above, to complement the *tyrR*-knockout strain, with the resulting strain named *A*. *brasilense* 2117. The correct sequence of the *tyrR* gene in pBSK-*tyrR-*Sp7 was confirmed via DNA sequencing at the Sequencing Unit at UNAM. The strain *A*. *brasilense* 2118 was constructed by transforming the plasmid pJB3Tc20 into *E*. *coli* S17.1 competent cells. The plasmid was subsequently transferred by conjugation to *A*.*brasilense* 2116 as previously described [8], to obtain the strain 2118, used as a negative control (S2 Fig). The strain *A*. *brasilense* 2119 was constructed by conjugation of *E*. *coli* S17.1(pAB2116) with *A*. *brasilense* 2112 as the recipient to obtain *A*. *brasilense* 2119, used to determine the expression of *ipdC-lacZ* under the control of *tyrR* minus by determination of β-galactosidase activity as previously described [8] (Table 1).

### Reverse transcription-PCR (RT-PCR)

Bacteria were grown on K-malate minimal medium at 30°C for 18 h and then were immediately frozen in liquid nitrogen after being harvested. Total RNA was extracted from cells using the CTAB total RNA isolation system (Promega, Corporation) with minor modifications. The primers designed for RT-PCR are listed in Table 2. The quality of the RNA samples was assessed by gel electrophoresis, and RNA concentrations were determined using an EON spectrophotometer system (BioTeK). cDNA was synthesized from total RNA using a Maxima First Strand cDNA Synthesis Kit (Thermo Scientific), which allows for high-capacity cDNA reverse transcription, following the manufacturer’s instructions. The *gyrA* gene was used as an endogenous control for internal normalization [21], and independent experiments were performed in triplicate.

### Determination of IAA production

To assess the IAA production, the strains were inoculated in 20 mL of liquid K-malate minimal medium supplemented with 100μg/mL of tryptophan at an initial OD_600_ of 0.01 in 120 mL flasks. The strains were cultured with shaking at 150 rpm and 30°C for 16, 24, 36, and 40 h. Auxin production was estimated by HPLC, using IAA (Sigma Chemical No. 87-51-4) as a standard as previously described [30]. The protein concentration was assessed by the Bradford method (Bradford reagent, Sigma-Aldrich No. B6916) following the manufacturer’s instructions. Data are reported as the results of three independent experiments with two biological replicates.

### β-galactosidase assay

β-galactosidase assay was performed with *A*. *brasilense* 2112 and 2119 strains grown in K-malate minimal medium containing 100 μg/mL of tryptophan with an inoculum of a 0.01 (OD_600_nm) and grown with shaking at 150 rpm and 30°C for 16, 24, and 36 h to obtain exponentially to stationary phase grown cultures. Then, 50 μL of cells were immediately harvested for assays. The β- galactosidase assays were carried out using 2-Nitrophenyl β-D-galactopyranoside (ONPG, 100 μL at 8 mg/mL; Sigma Chemical No. N1127) as the substrate, as previously described [8]. Data are reported as Miller units by mg of protein from three independent experiments with two biological replicates.

### Determination of bacterial growth curves

The growth of the *A*. *brasilense* Sp7, *A*. *brasilense* 2116, *A*. *brasilense* 2117, and *A*. *brasilense* 2118 strains was measured in K-malate minimal medium or K- minimal medium supplemented with 20 mM of DL-alanine, or D-alanine as carbon sources for each independent culture; or in K- malate minimal medium supplemented with 20 mM of DL-alanine as a nitrogen source. The bacterial cultures were incubated at 30°C for 36 or 58 h with shaking at 150 rpm. The growth of each strain was assessed by monitoring the OD_600_ at four-hour intervals. Three independent cultures were assayed for each experiment.

### Biofilm production

The biofilm production of the *A*. *brasilense* strains was assayed by growing bacteria in the NFb* modified nitrogen-free (C/N = 2 ratios; 27.6 mM malic acid and 10 mM D-alanine as an N source) medium at 30°C under static conditions, as previously described [31]. Briefly, bacteria were grown in CR plaques for 3 days at 30°C. Next, from each strain, a colony was inoculated into 12 mL of LB* medium (LB broth, 2.5 mM MgCl_2_; 2.5 mM CaCl_2_) in 50 mL flasks, and incubated at 30°C with shaking at 100 rpm for 16 h to achieve an OD_590_ nm of 1.1 to 1.4. The cells were harvested by centrifugation at 5000 rpm for 10 min, washed with phosphate buffer (66mM, pH 6.8) and resuspended to a final OD_590_ nm of 2. The cell suspensions were diluted 1:100 in NFb* modified medium. Two mL of cell suspensions were transferred to glass tubes and incubated under static conditions for 5 days at 30°C.

### Congo red assay and Calcofluor white colorant stain for exopolysaccharides (EPS) production

The EPS quantification by CR assay was performed as previously described [32], briefly the *A*. *brasilense* and derivative strains were grown in 125 mL flasks containing 25 ml of Nfb* medium and were incubated at 30°C under static conditions for five days at 30°C. Subsequently, from two mL of each bacterial culture the OD_600_ nm was determined and harvested by centrifugation at 10,000 rpm (Biofuge-Heraeus, Thermo Scientific) for 3 min and suspended in 2 mL NFb* medium to which a Congo red (CR) solution of 0.005% (w/v) (CR Sigma Aldrich, No. 22120) was added to achieve a 40 μg/mL concentration. The cells were incubated with shaking (200 rpm) for 2 h. The cultures were pelleted by centrifugation at 10,000 rpm. The amount of CR remaining in the supernatant was determined by measuring the OD_490_ nm of the solution and comparison with the appropriate CR concentration (10–300 μg/mL) to obtain mg CR. Independent experiments were performed by triplicate. CR binding was expressed as mg CR/OD_600_ nm. In addition, EPS production was determined by fluorescence of the *A*. *brasilense* Sp7, 2116 and 2117 colonies growing on Nfb* agar plates supplemented with 500 μg/mL of Calcofluor White Colorant M2R (CWC). Plates were incubated for 5 days at 30°C and then examined under UV light.

### Confocal Laser Scanner Microscopy (CLSM)

To assess the EPS staining with CWC the *A*. *brasilense* Sp7 and 2116 strains were grown in FluoroDish glass bottom dishes (fisher Scientific), as described above, and visualized using an inverted CLSM Nikon Eclipse Ti-E C2+ (Nikon Instruments Inc., Melville, New York) equipped with a 60x Plan Apo lambda objective. The biofilm was visually examined by differential interference contrast (DIC) and CLSM; CWC was excited with a 405-nm UV laser. The samples were scanned at an x/y scanning resolution of 1,024×1,024 pixels. Step size in z direction was 0.1 μm. The image stacks were visualized and processed using the software NIS Elements, Nikon. The images were edited by Image J software [33].

## Results

### Domains and structural characteristics of the TyrR protein from *A*. *brasilense* Sp7

**Fig 1:**
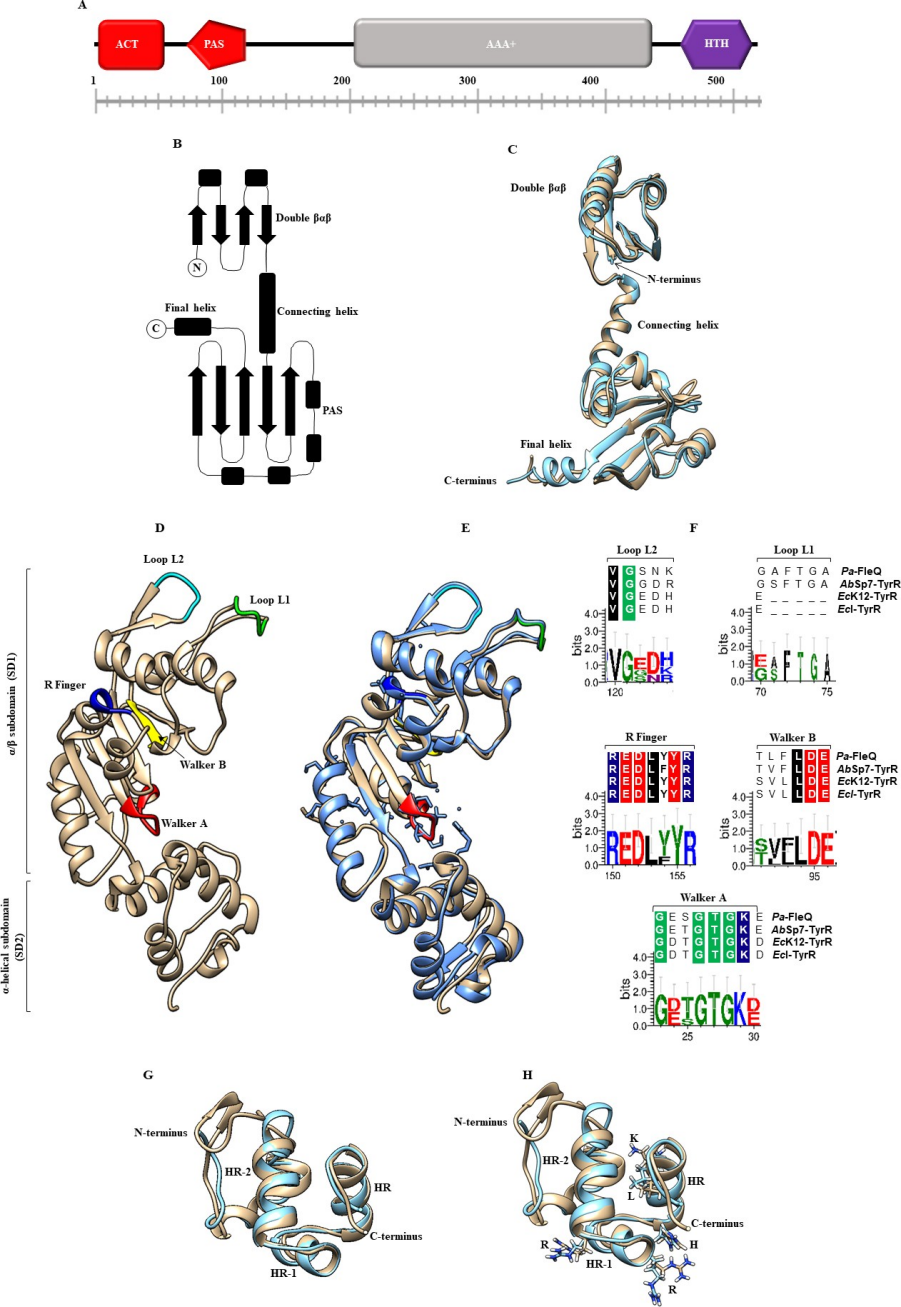
The overall predicted and domains displayed by the TyrR protein from *A*. *brasilense* Sp7 (Ab-Sp7). (**A**) The TyrR protein consists of an ACT domain (red square), a PAS domain (red pentagon), an AAA+ domain (gray rectangle) and an HTH motif (purple hexagon). (**B**) The topology of the N-terminal region of a TyrR monomer from *E*. *coli* K12 with its separate domains. (**C**) Comparison by superposition of monomers of TyrR from the *E*. *coli* K12 (blue) crystal structure with a resolution of 2.3 Å and Ab-Sp7 (beige) with a root mean square deviation (RMSD) of 1.59 Å. (**D**) The prediction of the AAA+ monomer domain of TyrR from Ab-Sp7. The ribbon representation of Ab_TyrR-AAA+ consists of an α/β sub domain followed by a smaller α-helical subdomain. The regions and the PAS domain are highlighted. (**E**) Superimposition of the Pa_FleQ-AAA+ crystal structure with a resolution of 1.8 Å (blue) and Ab_TyrR-AAA+ (beige) with a RSMD of 0.89 Å. The significant motifs are highlighted: Walker A (red), Walker B (yellow), R finger (dark blue), Loop L1, (green), Loop L2 (cyan). (**F**) Sequence logo representations of sequence alignments of Walker A (GXXXXGK), Walker B (hhhhDE), R finger (RXXXXXR), Loop L2 (VG), Loop L1 (GAFTGA), and multiple sequence alignments of FleQ (*P*. *aeruginosa*), TyrR (*A*. *brasilense* Sp7), TyrR (*E*. *coli* K12), TyrR (*E*. *cloacae*) were used. (**G**) Comparison by structure superimposition of the Ab-TyrR-HTH model (beige) and template 1G2H of *H*. *influenzae* (blue color). Three well defined α-helices (HR-2, HR-1, and HR) are shown. (**H**) Superimposed structures show the crucial amino acids involved in forming hydrogen bonds directly with DNA are highlighted: arginine 291 (R), arginine 296 (R), histidine 301 (H), lysine 307 (K), and leucine 308 (L). The homology models constructed by I-Tasser with the best scores, C-score -1.21, RMSD 1.25 Å, visualized with UCSF Chimera software. Logos were generated with the Weblogo 3 Create software.

**Fig 2:**
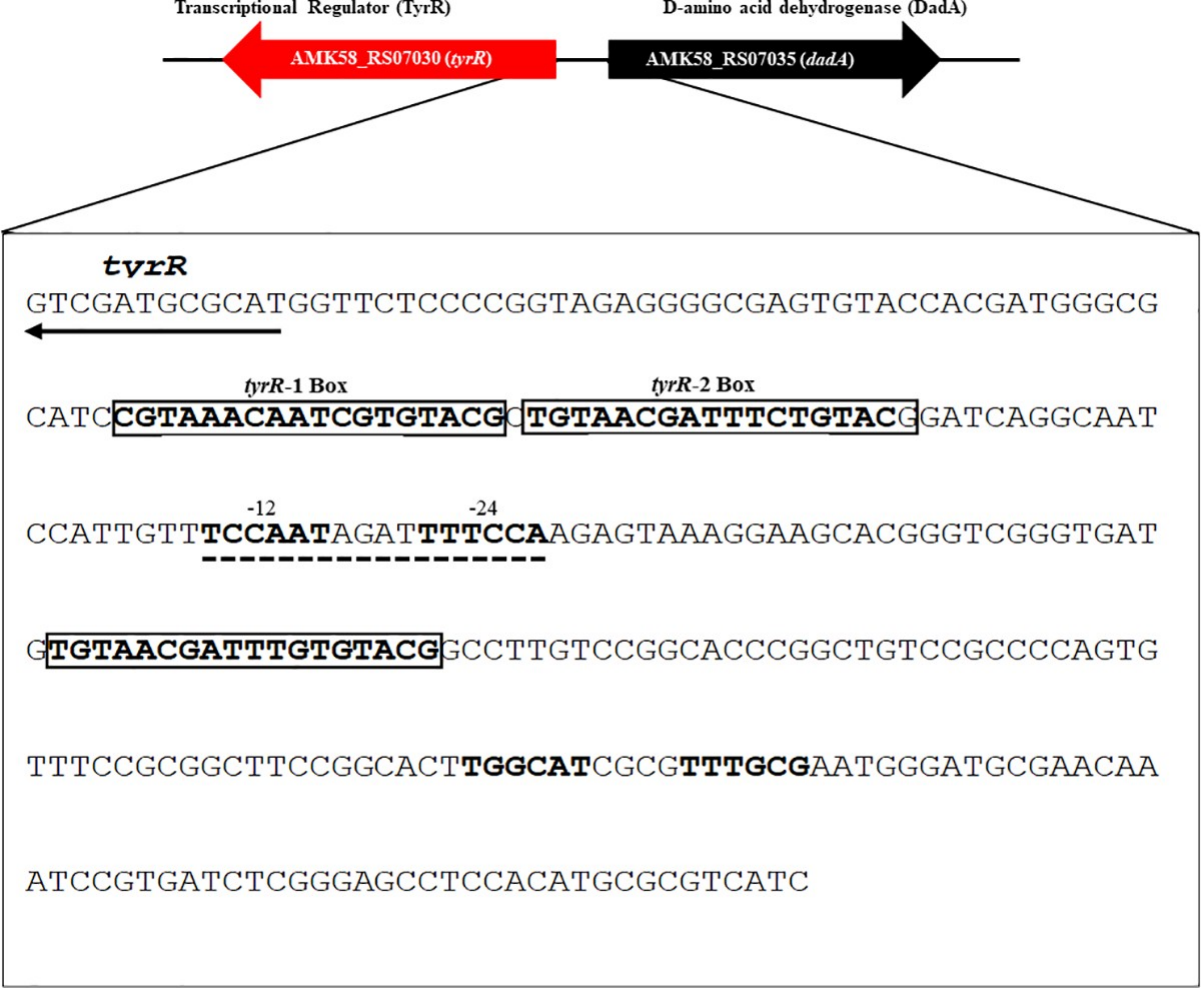
Sequences of the cis elements contained in the intergenic promoter region of the *tyrR* and *dadA* genes from *A*. *brasilense* Sp7. The TyrR boxes are shown in blod and are included in a rectangle; the -12 and -24 promoter regions are indicated in bold and with lines. The arrows indicate the ORFs of the *tyrR* and *dadA* genes. Identification of *tyrR* boxes in the *A*. *brasilense* Sp7 genome using the Find Individual Motif Occurrences (FIMO) software tool, which is part of the MEME Suite software toolkit [42].

The goals of this study were to clone the *tyrR* gene from *A*. *brasilense* Sp7, which encodes the transcriptional regulator TyrR, and determine if this protein is involved in IAA biosynthesis as previously described by Ryu and Patten [6]. We began with a bioinformatics BLASTP search using the TyrR amino acid sequence from *E*. *coli* K12 (GenBank accession number; NP_415839.1) against the *A*. *brasilense* Sp7 genome [34]. This search resulted in the identification of a protein encoded by the gene AMK58_RS07030 (GenBank accession number WP_035673475.1; E value: 4e-122) exhibiting a 43% identity and a 59% of similarity with the TyrR *E*. *coli* protein. The *A*. *brasilense* Sp7 *tyrR* gene was cloned and sequenced, and the translated protein was analyzed by multiple sequence alignment with TyrR proteins (S3 Fig). In addition, the TyrR protein was analyzed by structural studies using the N-terminus of the TyrR protein model from *E*. *coli* K12 [25] the central AAA**⁺** ATPase domain of Pa_FleQ-AAA+ from *P*. *aeruginosa* FleQ protein model [26], and the C-terminal DNA binding domain from the TyrR protein model from *H*. *influenzae* [27] (Fig 1). The putative protein shared a high similarity with each domain with respect to domain organization, signature motifs, secondary structure and topography characteristics that are exhibited by bacterial enhancer-binding proteins (eEBP), [35]. A comparison of the deduced amino acid sequence and a structural analysis among TyrR domain proteins indicates that TyrR contains an N-terminal PAS domain (**P**er-**A**RNT-**S**IM, residues 79 to 146) and an ACT domain (**A**spartate kinase, **C**horismate mutase, and **T**yrA) containing highly conserved aspartate (D) and arginine (R) amino acid residues. Both of these domains have been implicated in binding to aromatic amino acids and have been shown to participate in protein oligomerization and in modulating the activation or repression of gene expression (Fig 1A–1C). The *A*. *brasilense* Sp7 TyrR protein contains both a PAS domain and an ACT domain, as indicated by the superimposed models (Fig 1A–1C). In *E*. *coli* K12 TyrR protein, the PAS domain facilitates the transcriptional activation or repression of genes involved in aromatic amino acid biosynthesis and transport [25, 35]. The ACT domain is most likely the binding site for the aromatic amino acids tyrosine, phenylalanine, or tryptophan, whereas the PAS domain has been suggested to have a role in contacting the αCTD of RNAP [2, 25, 36]. The adjacent central domain contains a putative ATP binding site that was identified on the basis of sequence homology with the Walker A (GXXGXGKE) and Walker B (TVFLDE) ATP binding sites [26, 36]. This domain is responsible for ATP hydrolysis (Fig 1D–1F) and had features, such as the specific σ54 interaction loop-L1, which contains the signature GAFTGA motif that is indispensable, and often sufficient, to activate σ54-dependent transcription. This observation showed that the TyrR protein domains exhibits significant similarity to those of other regulators belonging to the bEBP bacterial superfamily of AAA**⁺** proteins, including NtrC/NifA [37] (Fig 1F and S3 Fig), FleQ from *P*. *aeruginosa* [26], and the TyrR proteins from *E*. *coli*, and *E*. *cloacae* [2, 6]. However, the *A*. *brasilense* TyrR completely differs from those of *E*. *coli* and *E*. *cloacae* in that it may regulate transcription at σ54-dependent promoters, as observed in *P*. *aeruginosa* [38], because of the presence of a highly conserved loop-L1. This feature is essential for contact with the σ54 subunit (Fig 1F and S3 Fig) but not with the σ70- dependent factor, as is observed in TyrR proteins from *E*. *coli* and *E*. *cloacae*, where the motif is missing (Fig 1B and S2 Fig), [4, 39]. These characteristics are similar to those observed for FleQ from *P*. *aeruginosa*, although differences are remarked in its N-terminus, which harbors a REC domain [26]. The C-terminus includes the helix-turn-helix (HTH) DNA-binding motif from amino acid residues 463 to 510 (Fig 1A, 1G and 1H), [2, 35, 40]. The HTH DNA binding domain structure consists of three well defined α-helices HR, HR-1, and HR-2, which share significant structural similarity to the catabolite activator protein (CAP) family in terms of the spatial orientation of the three helices [41]. In addition, the superimposed structures show that the crucial amino acids: arginine 291 (R), arginine 296 (R), histidine 301 (H), lysine 307 (K), and leucine 308 (L) involved in forming hydrogen bonds directly with DNA are present in the *A*. *brasilense* HTH domain (Fig 1H).

### Analysis of DNA sequences upstream of the *tyrR* gene

Next, a bioinformatics analysis was conducted using the software tool Find Individual Motif Occurrences (FIMO) [42]. The collection of TyrR-boxes from *E*. *coli* [2, 43], *E*. *cloacae* [6], and *Yersinia pestis* [44], each one possessing 100% identity in strong boxes, were used to scan the whole genome of the *A*. *brasilense* Sp7 strain [34] to identify DNA sequences that might potentially be bound by TyrR. We identified motifs bearing strong homology with those described as binding sites for TyrR (Fig 2 and S1 Table) [2, 6, 44] and include the consensus sequence (TGTAACG-N4-CTTTACA). In addition, this analysis provided valuable information regarding the putative TyrR boxes in the promoter region of genes that may be regulated by TyrR. As described by our data and by others [3, 5, 45], several regulons (amino acid metabolism, fatty acid metabolism, glucosyl transferase, signal transducing activity, membrane transport activity, and transcriptional regulation) as well as genes of unknown function have promoter sequences containing motifs that match with one or more TyrR boxes. A list of motif occurrences and genes that are predicted to be regulated by TyrR is shown in S1 Table.

Moreover, the DNA sequences adjacent to the *tyrR* gene were scrutinized for the presence of target sites for the TyrR protein. The consensus sequence based on TyrR boxes was used as the reference system, as described above. This was compared with those described for TyrR for the intergenic promoter region (Fig 2) indicating that the region possesses three TyrR boxes. Downstream of the *tyrR* gene, in divergent direction, we found the AMK58_RS07035 gene, which encodes for a putative D-amino acid dehydrogenase that is involved in D-alanine catabolism [46].

### Determination of IAA production and expression of the *ipdC-lacZ* transcriptional fusion

**Fig 3:**
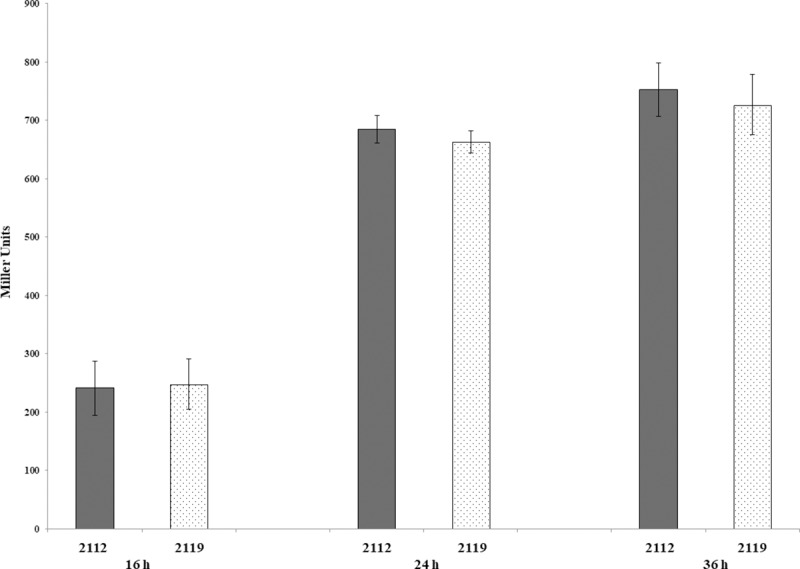
Expression analysis of *ipdC-lacZ* transcriptional fusion under the control of *A*. *brasilense* 2119 *tyrR* minus strain. *A*. *brasilense* strains were grown in minimal media (MM) with malate, as described in the Material and Methods section, during 16, 24 and 36 h. Data are reported as Miller units by mg of protein from three independent experiments with two biological replicates.

To determine the consequence of an insertional mutation in the *tyrR* gene, regarding IAA production and expression of the *ipdC* gene in the absence of the TyrR regulator, the supernatants of both the wild-type and *tyrR-*knockout mutant were evaluated for IAA production. IAA production was not significantly reduced after 16 to 40 h of growth (Table 3), indicating that unlike in *E*. *cloacae* [6], the Sp7 strain transcriptional regulator TyrR is not directly involved in regulating IAA biosynthesis. This result was confirmed by assaying an *ipdC-lacZ* transcriptional fusion [8] in the *tyrR-*knockout strain, and no change in β-galactosidase activity was observed (Fig 3).

**Table 3:** IAA production determination in *A*. *brasilense* wild-type strain and derivatives.

	AIA production μg/mg protein			
Strain	16 h^a^	24 h^b^	36 h^c^	40 h^d^
*A*. *brasilense* Sp7	6.38* (36.4 μM)	29.1* (166.1 μM)	37.4* (213.4 μM)	38.79* (221.4 μM)
*A*. *brasilense* 2116	3.84* (21.9 μM)	23.35* (133.2 μM)	30.66* (175 μM)	35.62* (203.3 μM)
*A*. *brasilense* 2112	ND	2.37* (13.5 μM)	4.05* (23.1 μM)	5.2* (26.6 μM)

* IAA production was determined at

^a^ exponential phase (OD_600_ = 1.04 ± 0.01)

^b^ initial stationary phase, (OD_600_ = 1.29 ± 0.01)

^C^ stationary phase, (OD_600_ = 1.34 ± 0.02); and

^d^ late stationary phase, (OD_600_ = 1.34 ± 0.01). The results are from three independent experiments. ND, not determined.

### TyrR positively regulates D-alanine utilization as a sole carbon and nitrogen source

**Fig 4:**
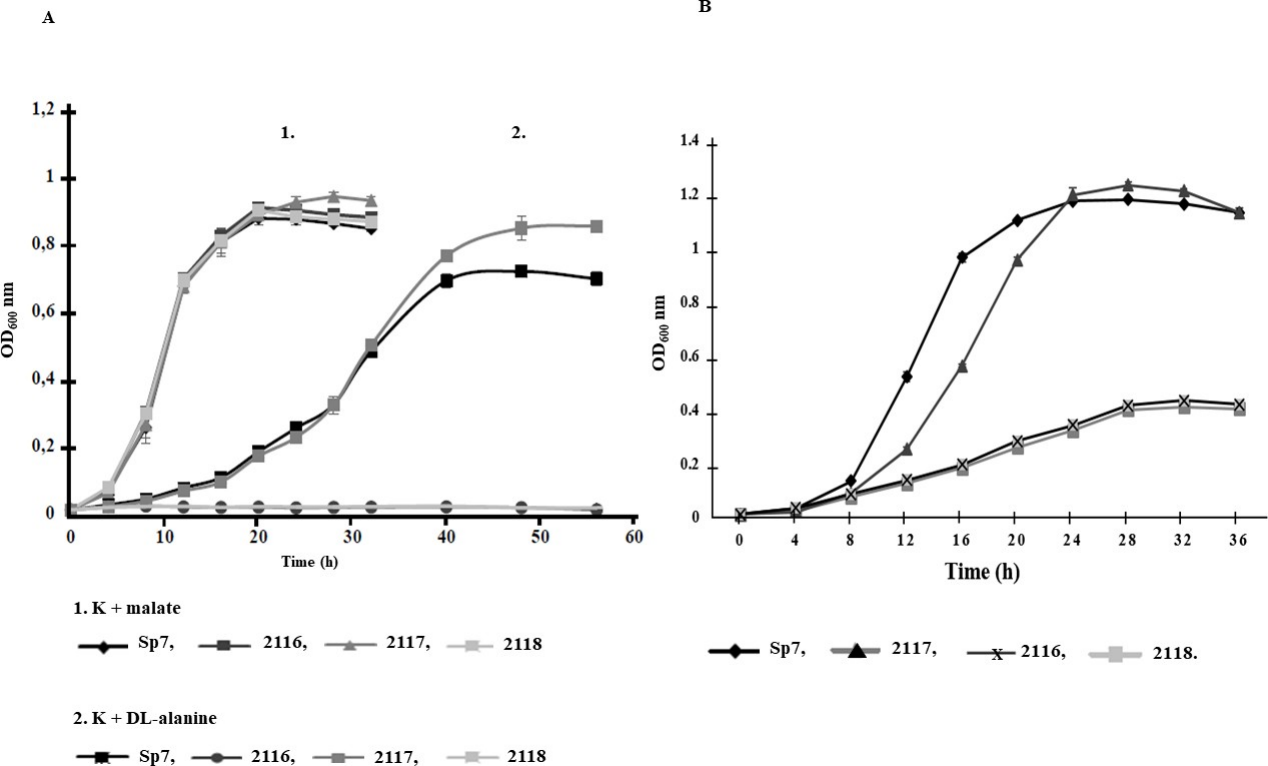
Growth profiles of the *A*. *brasilense* Sp7, 2116, 2117, and 2118 strains. (**A**) Growth kinetics of *A*. *brasilense* strains in minimal media (MM) with malate or D-alanine as the sole carbon sources. The strains were grown in MM supplemented with malate (20 mM) or DL-alanine (20 mM) as carbon sources. Growth was followed at OD_600_ nm every 4 h until 56 h. Sp7, 2116, 2117 and 2118 strains.The data points represent the average from three independent cultures of each strain. (**B**) Growth of *A*. *brasilense* strains in minimal medium with D-alanine as the sole nitrogen source. Bacterial strains were grown in DL-malate minimal medium supplemented with D-alanine (20 mM) as the sole nitrogen source. Growth was followed at OD_600_ nm every 4 h until 36 h. The data points represent the average from three independent cultures of each strain.

**Fig 5:**
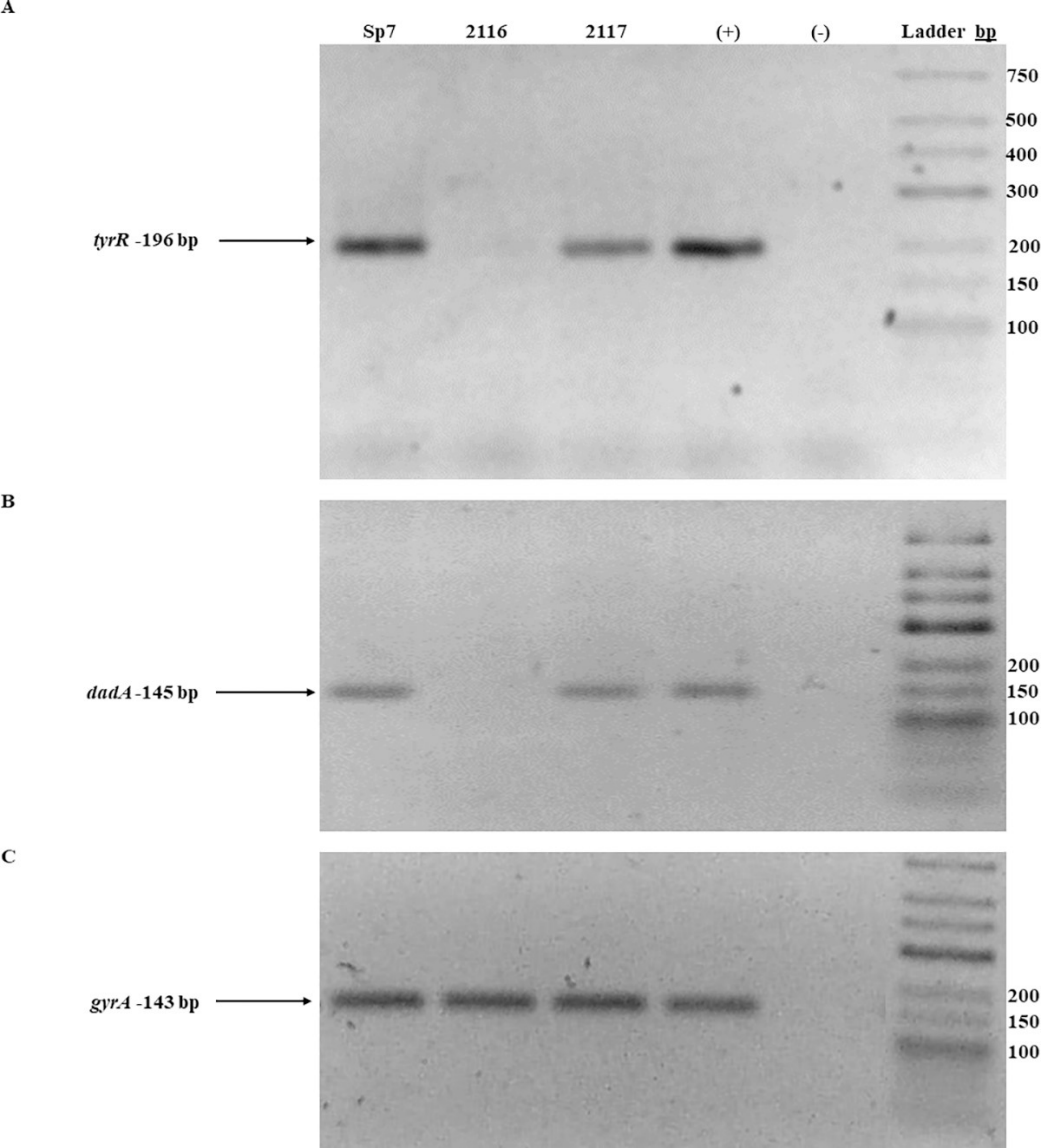
RT-PCR analysis of *dadA*, *tyrR* and *gyrA* expression from the *A*. *brasilense* Sp7, 2116, and 2117 strains. RT-PCR analysis of *tyrR*, *dadA* and *gyrA* expression from the *A*. *brasilense* Sp7, 2116 and 2117 strains. Total RNA (1 μg) from each strain was subjected to reverse transcription, and the obtained cDNA was amplified using pairs of primers specific for *tyrR*. (**A**) *dadA*, (**B**), and *gyrA* (**C**) *A*. *brasilense* strains are indicated at the top of each lane by their conventional denomination. The expected PCR products of 196 bp (*tyrR*), 145 bp (*dadA*) and 143 bp (*gyrA*) were visualized by ethidium bromide staining on a 2.5% agarose gel. A positive control (+), represented by genomic DNA. A negative reaction control is represented by (-), without reverse transcriptase, both were included in each experiment. The image is representative of three independents experiments.

The promoter region analysis predicted two putative boxes upstream of the *tyrR* gene and a third box downstream of the divergently transcribed gene *dadA*, encoding D-alanine dehydrogenase (Fig 2). Then, we were prompted to investigate if the *tyrR* mutation is involved in disrupting the catabolism of the amino acid D-alanine. The ability to utilize D-alanine as sole carbon (C) source was absolutely dependent upon the presence of an active putative D-alanine dehydrogenase, essential for the metabolism of D-alanine as sole C source (Fig 4A). This indicates that the *tyrR-*knockout mutant is unable to grow using this amino acid as a sole C source and that the *dadA* gene is positively regulated by TyrR (Fig 5). This result agrees with earlier data obtained for the *E*. *coli* K12 strain, where D-alanine was determined to be used as a C, nitrogen (N), and energy sources[46]. As the *A*. *brasilense* Sp7 *tyrR* mutation abolished growth in minimal medium with D-alanine as the sole C source, it was conceivable that TyrR plays a role in D-alanine utilization as a N source. To test this hypothesis, we examined the ability of the Sp7, 2116, 2117 and 2118 strains to grow in liquid DL-malate minimal medium (MM) without NH_4_Cl but supplemented with D-alanine (20 mM) as the sole N source. As shown in Fig 4B, a significantly reduced growth was obtained as a result of the mutation of the *tyrR* gene, whereas the wild-type Sp7 strain exhibited a similar growth pattern as in MM-malate. The growth of the 2116 mutant was completely restored to wild-type levels by reintroducing the *tyrR* gene *in trans*. However, the 2116 carrying the vector strain (2118 strain) did not restore growth, indicating that observed growth phenotype was due to the *tyrR* mutation. This result shows that the putative D-alanine dehydrogenase is required to some extent for D-alanine utilization as an N source in *A*. *brasilense*. However, lower growth was obtained in *A*. *brasilense* strains 2116 and 2118, compared to the wild-type Sp7 strain (Fig 4B). We suggest that this specific low growth rate may be due to D-amino acid oxidase (DAO) activity, because D-amino acids (D-AAs) can be catabolized via deamination, mainly in an oxidative way by D-AA oxidases, which have been used to demonstrate that are enzymes present in several bacteria [47].

Examining if TyrR regulates *dadA* expression, the presence of *dadA* mRNA was assessed by RT-PCR (Fig 5). The *dadA* mRNA was clearly missing in the mutant, compared to the wild-type. Therefore, TyrR appears to regulate DadA expression at the transcriptional level. When TyrR was expressed from a plasmid in the mutant strain, the *dadA* mRNA was restored to similar levels as the wild-type strain (Fig 5). This indicates that the protein encoded by *tyrR* regulates the transcription of *dadA* gene under the growth conditions assayed.

### Biofilm production

**Fig 6:**
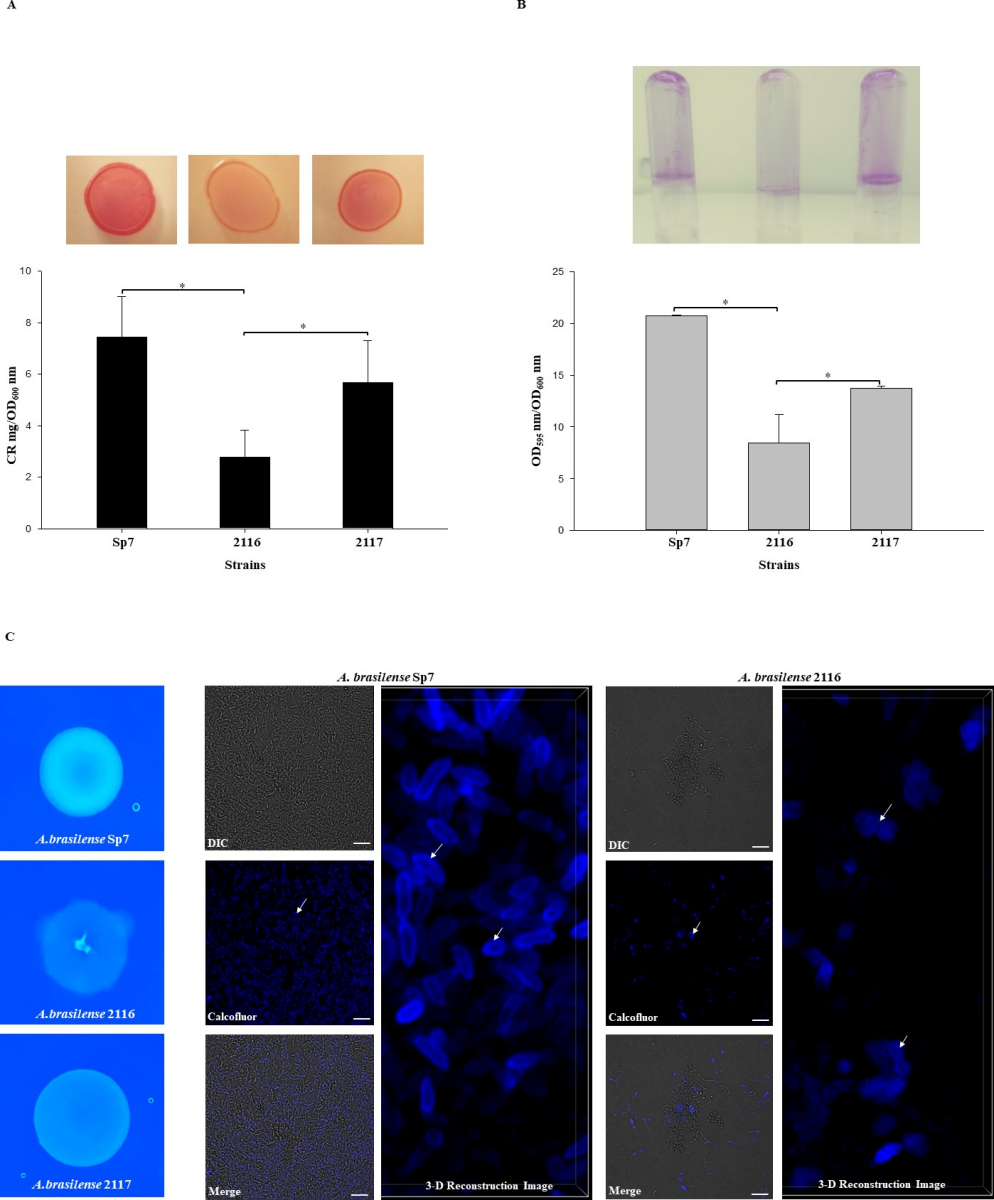
Biofilms and EPS production by the *A*. *brasilense* Sp7, 2116, and 2117 strains. (**A**) CR staining assay. Cells were grown on agar-solidified CR medium inoculated with 10 μL of 1.0 OD_600_ nm of each culture strains for 72 h at 30 ºC. For EPS quantification, the strains were cultured as described in Material and Methods and incubated at 30°C for 5 days. CR binding was expressed as mg CR/OD_600_ nm. (**B**) For biofilm production cells were grown statically for 5 days at 30 ºC on NFb* with 27mM malate as a C source + 10mM D-alanine. Biofilm formation was visualized by Crystal violet staining as well quantified and normalized per OD_600_ nm of growth. All data are the average from three independent experiments performed in duplicate. Error bars indicate standard errors of the means as compared with values observed for WT. For all data, *P* was < 0.001 as assessed by Student´s t-test. (**C**) Calcofluor white colorant M2R (CWC) staining. Cells were grown on agar-solidified NFb* supplemented with CWC and inoculated with 10 μL of 1.0 OD_600_ nm of each culture strains for five days at 30 ºC. The cell fluorescence was observed under was observed with a Nikon Eclipse Ti-E C2+. Bars represent 10 μm. The white arrows indicate EPS.

It was interesting to observe that colony growth of the *tyrR*::Gm^R^ mutant in CR medium had a different appearance than the wild-type strain, demonstrating that the uptake of CR or Calcofluor white colorant (CWC) by this strain was considerably decreased (Fig 6A and 6C). The colony color and morphology phenotype of the mutant on CR or CWC agar was restored to that of the wild-type strain by complementation with the *tyrR* gene *in trans* (Fig 6A). These data were confirmed by quantification of exopolysaccharides (EPSs) production using the test of binding to CR (Fig 6A) [32, 48]. Furthermore, the visualization by fluorescence microscopy and CLSM of EPSs produced by the strains stained with CWC confirmed the data obtained with the CR assay (Fig 6C). EPSs produced by *A*. *brasilense* are well known to bind CR, and EPSs are known to be associated with the biofilm formation and matrix structure of biofilms [13, 14, 48]. Indeed, biofilm phenotypes are associated with mutants that are defective in EPSs production or strains that overproduce these EPSs due to the role that they play in biofilm structure and their extensive involvement in matrix construction during biofilm formation[49, 50]. To gain insight into the biofilm production of the *A*. *brasilense* 2116 mutant and the complemented 2117 strain, both strains were growth under *in vitro* conditions as described in the Material and Methods section. Biofilm production was significantly decreased in the mutant, compared with the WT strain (Fig 6). Moreover, biofilm production was partially restored when the *tyrR* gene was complemented in *A*. *brasilense* 2016 strain. These results indicated that the transcriptional regulator TyrR is involved in the regulation of biofilm formation in *A*. *brasilense* Sp7. As the putative TyrR protein regulates the formation of biofilm, we hypothesized that it might be through the regulation of genes encoding specific proteins, such as a putative glucosyl transferase that belongs to family 2, which in many cases is responsible for the transfer of nucleotide-diphosphate sugars to substrates such as polysaccharides [51], putative signal-transducing proteins such as Methyl-accepting chemotaxis protein (MCP), or a putative regulator protein as shown in S1 Table. It is also possible that, as TyrR boxes were predicted to be present in several genes encoding for putative MCP, these genes are inactive in the *tyrR* mutant, impeding the bacteria from reaching the plant surface and forming biofilms [52].

## Discussion

In this study, a comparison of the structure of the putative TyrR protein encoded by the *tyrR* gene from *A*. *brasilense* Sp7 with other DNA-binding TyrR structure of transcription factors was performed. The putative TyrR protein exhibited high sequence homology with other DNA-binding TyrR identified in other bacteria. All of the examined features exhibited by the *A*. *brasilense* Sp7 TyrR protein classified it as a bacterial enhancer binding protein. This implies that it is a σ54-dependent activator with sufficient structural similarity and that it could be classified as a member of the group II subfamily of AAA**⁺** (ATPases associated with various cellular activities) proteins [34].

To gain insight into the effects of the *tyrR* mutation in *A*. *brasilense*, a number of phenotypes were investigated. The results demonstrated that TyrR: (i) affects the transcription of *dadA*, which allows for the use of D-alanine as the sole C source and the partial consumption of this amino acid as the sole N source; (ii) causes a decrease in EPSs, as determined by the uptake of CR and CWC colorants; and (iii) importantly, it affects biofilm formation.

L-amino acids (L-AAs) are well known to be essential in all kingdoms as building blocks of proteins, and their D-enantiomers are known to fulfill important functions in microbes. Indeed, the most prominent example of D-alanine utilization is described in bacteria that incorporate D-alanine and D-glutamic acid into their cell wall as structural elements and to protect it from proteases that degrade peptides made with L-amino acids. In addition, D-alanine may be essential for growth, biofilm formation and the biosynthesis of several important metabolites [53]. There is some evidence that plants are able to metabolize D-amino acids; D-amino acid oxidases and amino acid racemases have been reported in plants, but very little information exists on the ability of plants to take up and assimilate these amino acids through their roots [54]. However, it was demonstrated that *Arabidopsis thaliana* is able to produce exudates containing D-alanine, which may also be present in root secretions [55]. Furthermore, wheat plants have been shown to be able to take up and assimilate D-Ala as a nitrogen source [56]. In this instance, it may be enzymatically converted to L-amino acid by DAO, providing a pool of amino acids necessary for proteins synthesis and for the anaplerotic reactions of the Krebs cycle.

The development of bacterial biofilms on surfaces typically involves several stages that are likely to occur on the surfaces of plant roots. The initial stages of biofilm formation depend on bacterial motility, which enables free swimming bacteria to reach a suitable surface [52]. Consequently, the flagella act as motility organelles that help cells move to favorable habitats, while they can also act as adhesion factors that promote cell attachment to surfaces. Next, the bacteria adhere to the surface, irreversibly attach to it, form microcolonies and secrete EPSs that are required for the interactions of the cells with the surface, with other cells and with alternative matrix components to develop the complex architecture of the biofilm [57]. Previous studies have demonstrated that the response regulator protein FlcA controls the shift of *Azospirillum* from a vegetative state to cyst-like forms, both in culture and in association with plants. The *flcA* minus mutant also showed a lack of EPS material on the cell surface [13], and it was shown in this work that the *tyrR* mutant exhibited a similar phenotype as that observed in a *flcA* mutant. However, the mechanism involved in regulation of biosynthesis of EPS and biofilm formation appears completely different to that described in this study, since in a proteomic study it has been shown that the targets genes controlled by FlcA are involved in stress tolerance, carbohydrate metabolism, morphological transformation and nitrogen fixation [58].

## Conclusions

In this study we determined that the TyrR transcriptional regulator is not involved in IAA production in the plant growth promoting bacterium *A*. *brasilense* Sp7; however, it is absolutely required for *dadA* expression for the utilization of D-alanine as a C source, and its partial use as an N source, as well as in EPS production and the formation of biofilms. In general, root exudates are composed of a large variety of chemical compounds. Because D-amino acids, such as D-alanine, are common in bacteria and in the soil, we suggest that they may be more important as a source of N to plants than has previously been recognized and may play a role in plant–microbe interactions. Moreover, it was determined that TyrR plays a significant role in regulating EPS production and biofilm formation, which had not been described until now. However, more detailed studies are needed to identify the genes that are regulated by TyrR that are involved in EPS production and affect biofilm formation in *A*. *brasilense*. Future work will determine if D-Alanine binds to TyrR to regulates *dadA* transcription and how the HTH DNA binding motif of the putative TyrR protein functions to regulate the expression of its target genes, as well as whether TyrR acts in a typical manner as other bEBP regulators by binding to consensus upstream activating enhancer elements.

Contrary to *E*. *coli* [1, 2, 43] where is known that TyrR regulates the tyrosine biosynthesis genes, in this study, TyrR boxes in the regulatory region were not identified upstream genes involved in tyrosine biosynthesis as shown in S1 Table. Furthermore, the *A*. *brasilense* genome contains two additional homologous copies of *tyrR* like genes (AMK58_18895 and AMK58_15645 with 40% and 36% identity, respectively)[34], which likely could be involved in such regulation.

## Supporting information

S1 Fig Construction of the *A*. *brasilense* 2116 strain. A) Schematic representation of the chromosomal region of the *tyrR* gene from *A*. *brasilense* Sp7. The arrows indicate the localization and orientation of the *tyrR* and *dadA* genes and oligonucleotides used for the *tyrR* gene amplification. B) A map of pAB2116 plasmid derived from pSUP202. C) A map of the chromosomal region from *A*. *brasilense* 2116; Gm^R^ = gentamycin resistance cassette. D) An agarose gel electrophoresis gel analysis image used to assess the correct replacement of the *tyrR* gene by the insertional mutation *tyrR*::Gm^R^ for PCRs: lane 1, Molecular weight 1 kb; lane 2, amplicon obtained from genomic DNA of Sp7 using the TyrR-F1 and TyrR-R1.1 primers; lane 3, amplicon obtained from genomic DNA of 2116 using the TyrR-F1 and TyrR-R1.1 primers; lane 4, amplicon obtained from genomic DNA from Sp7 using the primers PrTyrR-F-Sp7 and PrTyrR-R-Sp7; lane 5, amplicon obtained from genomic DNA from strain 2116 using the PrTyrR-F-Sp7 and PrTyrR-R-Sp7 primers; lane 6, PCR negative control using genomic DNA from Sp7 using the TyrR-F1 and RGm3 primers; lane 7, amplicon obtained using genomic DNA from strain 2116 using the TyrR-F1 and RGm3 primers. The amplicon size is indicated in bp.
(TIF)

S2 Fig Construction of the pJB3-*tyrR*-Sp7 plasmid for genetic complementation. A) Schematic representation of pJB3-*tyrR*-Sp7 harboring the *tyrR* gene, which is a derivative of the pJB3Tc20 plasmid [20]. Ap^R^ = Ampicillin resistance cassette; *tetA* and *tetR* genes encode for tetracycline resistance. B) An agarose gel electrophoresis image of the PCR amplicon of the native promoter and *tyrR* gene. C) An agarose gel electrophoresis image is showing the pJB3-*tyrR*-Sp7 plasmid DNA enzymatic digestion pattern with *Bam*HI, *Sal*I and *Nru*I restriction enzymes. D. Photography of agarose gel electrophoresis is indicating the PCR obtained from genomic DNA of Sp7, 2116 and 2118 strains, with PrTyrR-F-Sp7 and PrTyrR-R-Sp7 primers. Lane 1, 1 kb molecular weight marker; lane 2, amplicon obtained from genomic DNA of Sp7; lane 3, amplicon obtained from genomic DNA of 2116; lane 4; amplicon obtained from genomic DNA of 2118. The amplicon size is indicated in bp.
(TIF)

S3 Fig The domains and sequence alignment of the TyrR protein. A) Schematic representation and general architecture of the TyrR protein showing the N-terminus with a PAS domain comprising 79 to 146 amino acid residues; a central AAA+ domain comprising 221–366 residues, and in the C-terminus comprising 463–510 residues. B) The deduced amino acid sequence of the *A*. *brasilense* TyrR protein was aligned with those of the TyrR proteins from *E*. *coli* MG1165 (TyrR-Ec, NP_415839.1), TyrR from *E*. *cloacae* (TyrR-Ecl, WP_003856887.1), and GcsR from *P*. *aeruginosa* PAO1 (GcsR-Pa, NP_251139.1). The black box indicates residues that are highly conserved in all four TyrR proteins, and the gray font indicates positions at which only conservative changes have occurred. The red amino acid residues (D and R) are involved in the binding to aromatic amino acids. The red colored boxes indicate Walker A (I) and Walker B (III) motifs, which are ATP binding sites; sigma 54 (II) motif, and the HTH, DNA binding motif (IV). The *A*. *brasilense* and *P*. *aeruginosa* TyrR proteins possessed nine extra amino acid residues between the two ATP-binding motifs (A and B), which is suggested to be the σ54 binding site. The sequences in the HTH motifs are highly conserved in all four proteins. Clustal Omega (http://www.ebi.ac.uk/Tools/msa/clustalo/) [59]. Multiple Align Show (http://www.bioinformatics.org/sms/index.html).
(TIF)

S1 Table *A*. *brasilense* Sp7 genes with predicted TyrR boxes in their putative promoter regions. TyrR-boxes from *E*. *coli* [2], *E*. *cloacae* [6], and *Y*. *pestis* [44] were used to identify potential TyrR boxes in the *A*. *brasilense* Sp7 genome using the Find Individual Motif Occurrences (FIMO) software tool, which is part of the MEME Suite software toolkit [42]. Biological processes were inferred by comparison with homologous proteins in other bacteria using the Universal Protein Resource (UniProt) (http://www.uniprot.org/) Dist**, distance between the predicted TyrR boxes and the annotated translational start sites (number of nucleotides). ***Consensus sequence was generated with WebLogo application [29] using the predicted TyrR boxes.
(DOCX)
