# Supplementary figures and images for: TyrR is involved in the transcriptional regulation of biofilm formation and D-alanine catabolism in Azospirillum brasilense Sp7
Source: PLoS One. 2019 Feb 14;14(2):e0211904. doi: 10.1371/journal.pone.0211904 (PMC6375630; doi:10.1371/journal.pone.0211904)

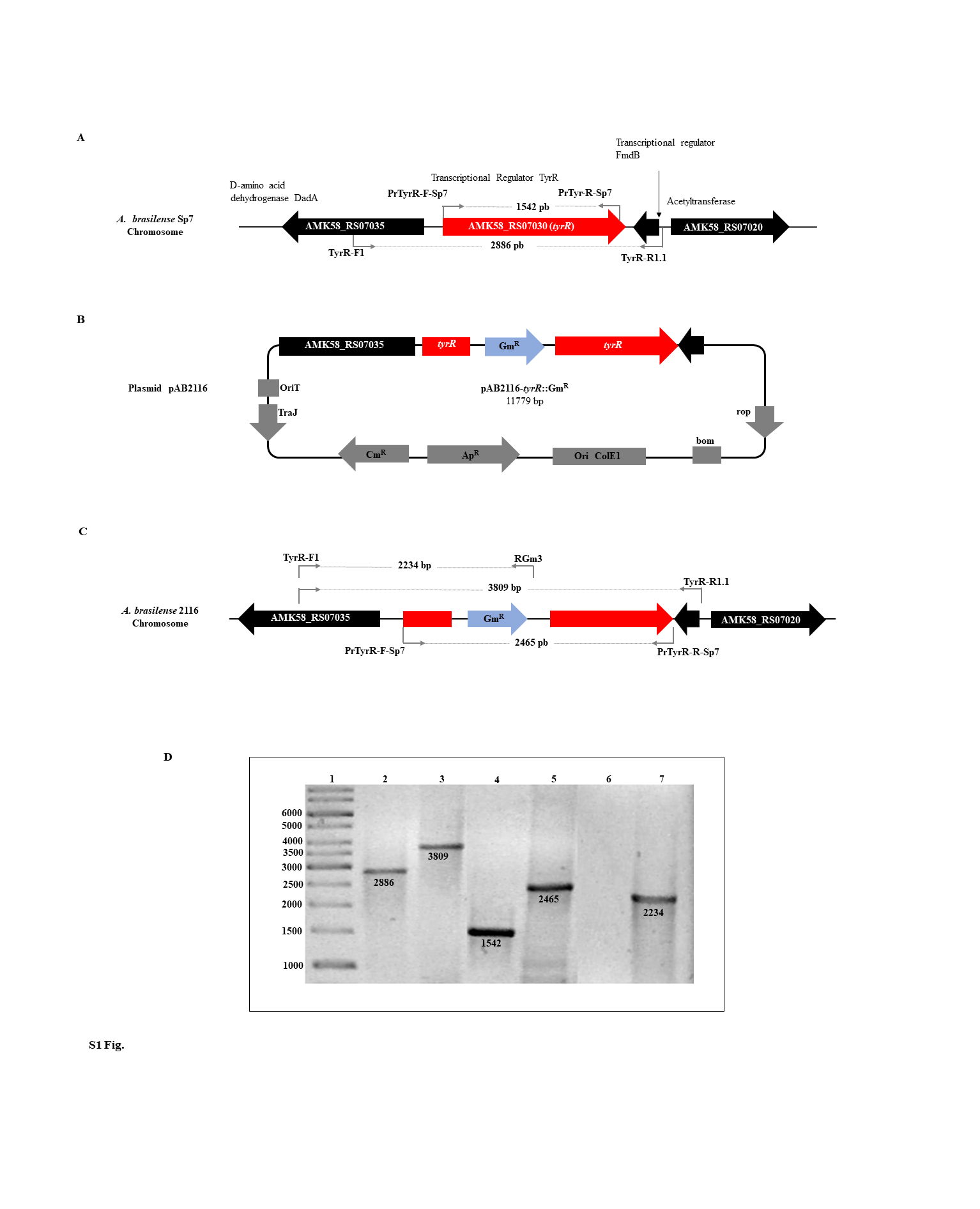

Supplement: S1 Fig — A) Schematic representation of the chromosomal region of the tyrR gene from A. brasilense Sp7. The arrows indicate the localization and orientation of the tyrR and dadA genes and oligonucleotides used for the tyrR gene amplification. B) A map of pAB2116 plasmid derived from pSUP202. C) A map of the chromosomal region from A. brasilense 2116; GmR = gentamycin resistance cassette. D) An agarose gel electrophoresis gel analysis image used to assess the correct replacement of the tyrR gene by the insertional mutation tyrR::GmR for PCRs: lane 1, Molecular weight 1 kb; lane 2, amplicon obtained from genomic DNA of Sp7 using the TyrR-F1 and TyrR-R1.1 primers; lane 3, amplicon obtained from genomic DNA of 2116 using the TyrR-F1 and TyrR-R1.1 primers; lane 4, amplicon obtained from genomic DNA from Sp7 using the primers PrTyrR-F-Sp7 and PrTyrR-R-Sp7; lane 5, amplicon obtained from genomic DNA from strain 2116 using the PrTyrR-F-Sp7 and PrTyrR-R-Sp7 primers; lane 6, PCR negative control using genomic DNA from Sp7 using the TyrR-F1 and RGm3 primers; lane 7, amplicon obtained using genomic DNA from strain 2116 using the TyrR-F1 and RGm3 primers. The amplicon size is indicated in bp. (TIF) [file pone.0211904.s001.tif]

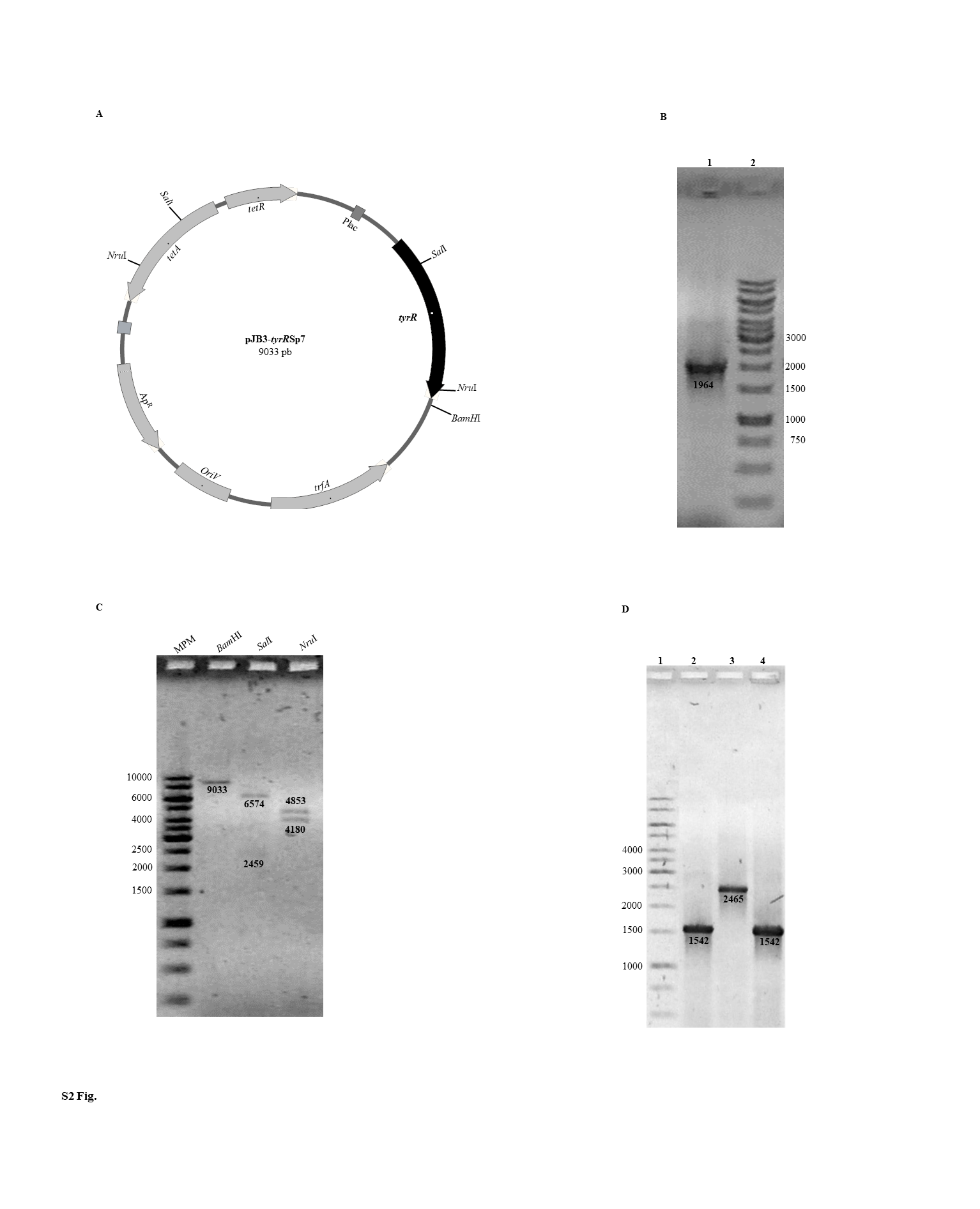

Supplement: S2 Fig — A) Schematic representation of pJB3-tyrR-Sp7 harboring the tyrR gene, which is a derivative of the pJB3Tc20 plasmid [20]. ApR = Ampicillin resistance cassette; tetA and tetR genes encode for tetracycline resistance. B) An agarose gel electrophoresis image of the PCR amplicon of the native promoter and tyrR gene. C) An agarose gel electrophoresis image is showing the pJB3-tyrR-Sp7 plasmid DNA enzymatic digestion pattern with BamHI, SalI and NruI restriction enzymes. D. Photography of agarose gel electrophoresis is indicating the PCR obtained from genomic DNA of Sp7, 2116 and 2118 strains, with PrTyrR-F-Sp7 and PrTyrR-R-Sp7 primers. Lane 1, 1 kb molecular weight marker; lane 2, amplicon obtained from genomic DNA of Sp7; lane 3, amplicon obtained from genomic DNA of 2116; lane 4; amplicon obtained from genomic DNA of 2118. The amplicon size is indicated in bp. (TIF) [file pone.0211904.s002.tif]

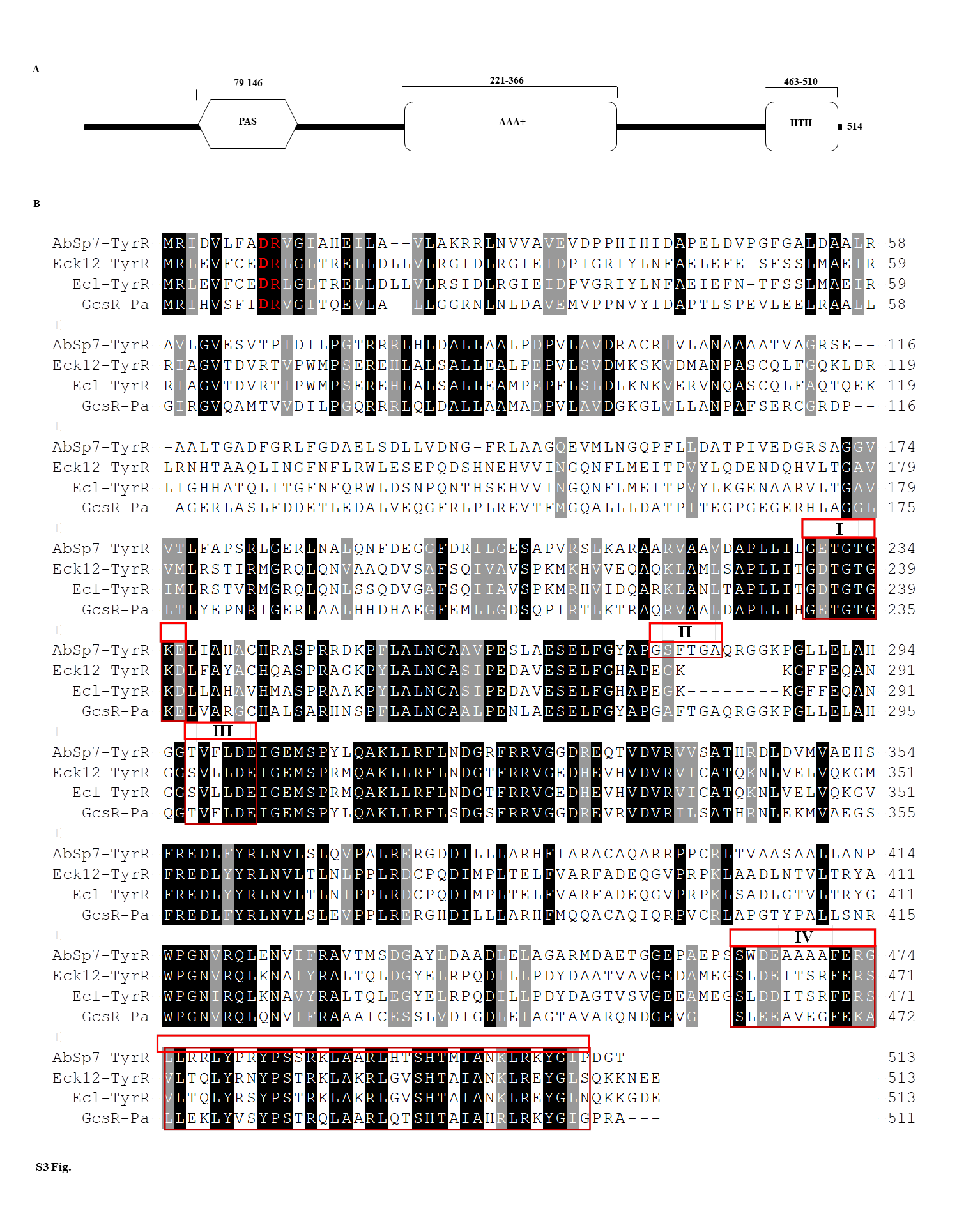

Supplement: S3 Fig — A) Schematic representation and general architecture of the TyrR protein showing the N-terminus with a PAS domain comprising 79 to 146 amino acid residues; a central AAA+ domain comprising 221–366 residues, and in the C-terminus comprising 463–510 residues. B) The deduced amino acid sequence of the A. brasilense TyrR protein was aligned with those of the TyrR proteins from E. coli MG1165 (TyrR-Ec, NP_415839.1), TyrR from E. cloacae (TyrR-Ecl, WP_003856887.1), and GcsR from P. aeruginosa PAO1 (GcsR-Pa, NP_251139.1). The black box indicates residues that are highly conserved in all four TyrR proteins, and the gray font indicates positions at which only conservative changes have occurred. The red amino acid residues (D and R) are involved in the binding to aromatic amino acids. The red colored boxes indicate Walker A (I) and Walker B (III) motifs, which are ATP binding sites; sigma 54 (II) motif, and the HTH, DNA binding motif (IV). The A. brasilense and P. aeruginosa TyrR proteins possessed nine extra amino acid residues between the two ATP-binding motifs (A and B), which is suggested to be the σ54 binding site. The sequences in the HTH motifs are highly conserved in all four proteins. Clustal Omega (http://www.ebi.ac.uk/Tools/msa/clustalo/) [59]. Multiple Align Show (http://www.bioinformatics.org/sms/index.html). (TIF) [file pone.0211904.s003.tif]
