# Supplementary material for: TyrR is involved in the transcriptional regulation of biofilm formation and D-alanine catabolism in Azospirillum brasilense Sp7
Source: PLoS One. 2019 Feb 14;14(2):e0211904. doi: 10.1371/journal.pone.0211904 (PMC6375630; doi:10.1371/journal.pone.0211904)
Supplement: S1 Table — TyrR-boxes from E. coli [2], E. cloacae [6], and Y. pestis [44] were used to identify potential TyrR boxes in the A. brasilense Sp7 genome using the Find Individual Motif Occurrences (FIMO) software tool, which is part of the MEME Suite software toolkit [42]. Biological processes were inferred by comparison with homologous proteins in other bacteria using the Universal Protein Resource (UniProt) (http://www.uniprot.org/) Dist**, distance between the predicted TyrR boxes and the annotated translational start sites (number of nucleotides). ***Consensus sequence was generated with WebLogo application [29] using the predicted TyrR boxes. (DOCX) [file pone.0211904.s004.docx]

**S1 Table.** *A. brasilense* Sp7 genes with predicted TyrR boxes in their putative promoter regions.

| **Locus tag/ cellular process^*^** | **Function** | **TyrR box sequence** | **P-value** | **Dist^**^** | **Localization** |
| --- | --- | --- | --- | --- | --- |
|  |  |  |  |  |  |
| **D-amino acid catabolic process** |  |  |  |  |  |
| AMK58_RS00180 | Fumaryl acetoacetate hydrolase | **CGTTA**CGCATTGGT**TCCG** | 6.73e-05 | 268 | Chromosome |
| AMK58_RS07035 | D-amino acid dehydrogenase DadA | **TGTAAC**GATTTCTG**TACG** | 5.6e-08 | 191 | Chromosome |
|  |  | **TGTAAC**GATTTGTG**TACG** | 8.91e-08 | 109 |  |
|  |  | **CGTAA**ACAATCGTG**TACG** | 9.99e-06 | 210 |  |
|  |  |  |  |  |  |
| **Amino acid biosynthetic process** |  |  |  |  |  |
| AMK58_RS12605 | Phosphoribosyl anthranilate isomerase | **TGTAA**GGACCTGTT**CCCA** | 7.12e-05 | -1 | Chromosome |
| AMK58_RS06400 | Anthranilate phosphoribosyl transferase | **TGGAA**AACTTCCTG**AACA** | 8.02e-05 | 25 | Chromosome |
|  |  |  |  |  |  |
| **Signal transducer activity** |  |  |  |  |  |
| AMK58_RS01275 | Response regulator | **TGGAA**TGTTTTCTT**TCCA** | 8.66e-07 | 293 | Chromosome |
| AMK58_RS10265 | PAS domain-containing sensor histidine kinase | **CGTCA**TGAATCGTT**AACG** | 5.9e-05 | 84 | Chromosome |
| AMK58_RS03745 | Histidine kinase | **TGTAT**TGCAGAATT**GACA** | 3.43e-05 | 181 | Chromosome |
| AMK58_RS03835 | Chemotaxis protein CheY | **GGTAA**CCCGATATT**TACG** | 4.81e-05 | 151 | Chromosome |
| AMK58_RS02000 | Chemotaxis phosphatase CheZ | **TGTAAC**CTCAGTTT**TCCT** | 3.21e-05 | 57 | Chromosome |
| AMK58_RS04505 | Methyl-accepting chemotaxis protein | **TGTAG**GGTATATTT**AACA** | 5.22e-05 | 63 | Chromosome |
| AMK58_RS04510 | Methyl-accepting chemotaxis protein | **TGGAA**TACAACCGT**TACT** | 7.3e-05 | 321 | Chromosome |
| AMK58_RS23860 | Methyl-accepting chemotaxis protein | **TGTCAA**TCTATATG**AACA** | 5.3e-05 | 204 | ABSp7_p2 |
| AMK58_RS04755 | Adenylate/guanylate cyclase domain-containing protein | **TGGAA**CATCGGCTG**TACG** | 5.26e-05 | 46 | Chromosome |
|  |  |  |  |  |  |
| **Transmembrane transporter activity** |  |  |  |  |  |
| AMK58_RS02255 | sn-glycerol-3-phosphate ABC transporter substrate-binding protein UgpB | **CGTCA**CCAAATCTT**CACA** | 4.42e-05 | 77 | Chromosome |
| AMK58_RS08700 | Lysine transporter LysE | **TGGAA**CGCTTTCTG**TCCG** | 8.47e-06 | 116 | Chromosome |
| AMK58_RS18435 | MlaE family lipid ABC transporter permease subunit | **TGAAA**AGGTTCCTG**TACA** | 3.92e-05 | 119 | ABSp7_p1 |
| AMK58_RS19935 | Na/Pi cotransporter family protein | **TGTTAC**GTTTTCAT**GACG** | 8.33e-05 | 66 | ABSp7_p1 |
| AMK58_RS22560 | Autotransporter domain-containing protein | **TGCAA**TTTAGTTTT**TACT** | 8.05e-05 | 115 | ABSp7_p2 |
| AMK58_RS14355 | Ferrous iron transport protein A | **CGTTAC**GCTTATTT**TACA** | 4.98e-05 | 116 | ABSp7_p1 |
| AMK58_RS23865 | Manganese efflux pump | **TGTTCA**TATAGATT**GACA** | 5.74e-05 | 229 | ABSp7_p2 |
|  |  |  |  |  |  |
| **Transcription regulation** |  |  |  |  |  |
| AMK58_RS07030 | TyrR transcriptional regulator | **CGTAC**AGAAATCGT**TACA** | 5.72e-07 | 63 | Chromosome |
|  |  | **CGTAC**ACAAATCGT**TACA** | 2.74e-06 | 145 |  |
|  |  | **CGTAC**ACGATTGTT**TACG** | 7.82e-06 | 44 |  |
| AMK58_RS15670 | Sigma-54-dependent Fis family transcriptional regulator | **TGGAAT**TTATTCAT**TCCA** | 3.09e-05 | 364 | ABSp7_p1 |
| AMK58_RS17410 | TetR/AcrR family transcriptional regulator | **TGTAAT**GTCCTGAT**TCCA** | 5.87e-05 | 229 | ABSp7_p1 |
|  |  |  |  |  |  |
| **Lipopolysaccharide biosynthetic process** |  |  |  |  |  |
| AMK58_RS28995 | Lipopolysaccharide biosynthesis protein | **TGGAA**AAATTTCTT**GACT** | 1.45e-05 | 507 | ABSp7_p5 |
|  |  |  |  |  |  |
| **Fatty acid metabolic process** |  |  |  |  |  |
| AMK58_RS27315 | Long-chain fatty acid--CoA ligase | **CGTAA**TTTTTTCTT**CACA** | 7.49e-06 | 149 | ABSp7_p3 |
|  |  |  |  |  |  |
| **Nucleotide biosynthesis** |  |  |  |  |  |
| AMK58_RS27345 | Ribose-phosphate pyrophosphokinase | **CGTCA**TGGAACGTT**TACA** | 2.72e-05 | 105 | ABSp7_p3 |
| AMK58_RS15665 | Thymidylate synthase | **TGGAAT**GAATAAAT**TCCA** | 7.49e-05 | 57 | ABSp7_p1 |
|  |  |  |  |  |  |
| **Protein N-linked glycosylation** |  |  |  |  |  |
| AMK58_RS12430 | Glycosyltransferase family 2 protein | **TGGAC**AGCTACCTG**TCCG** | 5.47e-05 | 103 | Chromosome |
|  |  |  |  |  |  |
| **Nitrate assimilation** |  |  |  |  |  |
| AMK58_RS01665 | Oxidoreductase | **TGAAC**GGATTTCGA**TACG** | 9.66e-05 | 113 | Chromosome |
|  |  |  |  |  |  |
| **Defense response to virus** |  |  |  |  |  |
| AMK58_RS11265 | Type I-C CRISPR-associated protein Cas5 | **CGTAA**AGATATTGG**CACG** | 9.06e-05 | 416 | Chromosome |
|  |  |  |  |  |  |
| **Unknown function** |  |  |  |  |  |
| AMK58_RS26970 | Hypothetical protein | **TGTAA**AGCAACCTT**GACA** | 6.2e-07 | 60 | ABSp7_p3 |
| AMK58_RS09155 | Hypothetical protein | **TGTAA**CGTGTTCAT**AACA** | 5.77e-05 | 91 | Chromosome |
| AMK58_RS16495 | Hypothetical protein | **TGGAC**CCATTCGGT**TCCG** | 8.21e-05 | 102 | ABSp7_p1 |
| AMK58_RS03155 | Hypothetical protein | **CGGAA**TCCCGTCTT**TACG** | 6.8e-05 | 120 | Chromosome |
| AMK58_RS02955 | PRC-barrel domain containing protein | **TGTCAA**GAATATTT**TCCT** | 7.61e-05 | 124 | Chromosome |
| AMK58_RS00460 | FxsA family protein | **TGTACC**GGAACGGT**GCCG** | 8.16e-05 | 134 | Chromosome |
| AMK58_RS07195 | Pentapeptide repeat-containing protein | **CGGAAC**GTCATGTT**AACA** | 4.64e-05 | 97 | Chromosome |
| AMK58_RS11295 | Calcium-binding protein | **TGTAT**AGTTAAAGT**CACA** | 4.64e-05 | 64 | Chromosome |
| AMK58_RS06745 | OmpA family protein | **TGTCA**CGTTACATG**CACG** | 8.02e-05 | 77 | Chromosome |
| AMK58_RS00280 | Membrane protein | **TGAAA**AGATTGATT**TCCG** | 1.04e-05 | 43 | Chromosome |
| Consensus sequence^***^ | 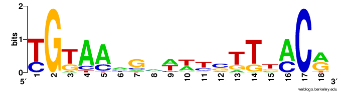 |  |  |  |  |
